# EEG source imaging of hand movement-related areas: an evaluation of the reconstruction and classification accuracy with optimized channels

**DOI:** 10.1186/s40708-024-00224-z

**Published:** 2024-05-04

**Authors:** Andres Soler, Eduardo Giraldo, Marta Molinas

**Affiliations:** 1 Department of Engineering Cybernetics, Norwegian University of Science and Technology, Trondheim, Norway; 2 Department of Electrical Engineering, Universidad Tecnológica de Pereira, Pereira, Colombia

**Keywords:** EEG, Source imaging, Channel optimization, Low-density EEG, BCI, Motor imagery, Classification

## Abstract

The hand motor activity can be identified and converted into commands for controlling machines through a brain-computer interface (BCI) system. Electroencephalography (EEG) based BCI systems employ electrodes to measure the electrical brain activity projected at the scalp and discern patterns. However, the volume conduction problem attenuates the electric potential from the brain to the scalp and introduces spatial mixing to the signals. EEG source imaging (ESI) techniques can be applied to alleviate these issues and enhance the spatial segregation of information. Despite this potential solution, the use of ESI has not been extensively applied in BCI systems, largely due to accuracy concerns over reconstruction accuracy when using low-density EEG (ldEEG), which is commonly used in BCIs. To overcome these accuracy issues in low channel counts, recent studies have proposed reducing the number of EEG channels based on optimized channel selection. This work presents an evaluation of the spatial and temporal accuracy of ESI when applying optimized channel selection towards ldEEG number of channels. For this, a simulation study of source activity related to hand movement has been performed using as a starting point an EEG system with 339 channels. The results obtained after optimization show that the activity in the concerned areas can be retrieved with a spatial accuracy of 3.99, 10.69, and 14.29 mm (localization error) when using 32, 16, and 8 channel counts respectively. In addition, the use of optimally selected electrodes has been validated in a motor imagery classification task, obtaining a higher classification performance when using 16 optimally selected channels than 32 typical electrode distributions under 10–10 system, and obtaining higher classification performance when combining ESI methods with the optimal selected channels.

## Introduction

The human primary motor cortex (M1) has been identified as the area responsible for commanding the execution of hand movements [23]. This area is characterized for exhibiting mainly a *mu* rhythm (frequencies around 8–12 Hz) at rest. An attenuation of the power of this rhythm, also called event-related desynchronization (ERD), in the contralateral cortex is presented during the execution/imagination of hand movements [22, 23]. This particular phenomenon in the *mu* rhythm has been exploited by brain-computer-interfaces (BCIs) to discern the hand that was executing an actual or imagined movement and convert those motor events into commands for a human peripheral system [9, 10, 24].

Most of the BCIs are based on the analysis performed using the information registered by the electrodes on the scalp (electrode space) [15], which is characterized by the low spatial resolution due to the volume conduction effect. In this, the potential generated by the electrical activity in the brain gets mixed and attenuated due to the different layers and their different conductivity properties before reaching the scalp. EEG Source imaging (ESI) methods can accurately retrieve the source activity and unmix the signals registered at the scalp; resulting in a better spatial discrimination of the underlying activity [10]. However, ESI requires high-density EEG (hdEEG) and a volume conduction model of the head, to perform accurate estimations [17]. Those requirements, in addition to computational concerns, might have contributed to fewer implementations of BCI systems based on source activity. Despite this concern, multiple studies have demonstrated that source-centered BCIs are feasible in online scenarios [2, 16] and can outperform the electrode only based BCIs [3, 7, 28]. However, ldEEG is still preferable in BCIs due to its lower cost, increased wearability, and ease of use.

Regarding the use of ldEEG in ESI, a recent study [26], presented an automated framework for optimal selection of ldEEG electrode positions that attained higher spatial accuracy than coverage-based electrode distribution and close to hdEEG accuracy. In [14], the authors used ldEEG, 26 channels, and source space to detect lower limb movements. Although ldEEG was utilized, no optimal electrode selection was conducted and electrodes were placed based on scalp coverage criteria. Inspired by those, here we propose an evaluation of the reconstruction accuracy with optimized channels with the purpose of exploring the boundaries of ldEEG for estimating the source activity of hand movement-related areas. To perform such evaluation, first, we simulated source activity in the region of interest (ROI). Then, we applied the framework of optimal selection of electrode location from [26] and introduced new constraints to evaluate the performance of symmetrical and non-symmetrical electrode distributions. The contribution of this paper is to conduct an evaluation of how accurate can the estimation of the source activity be in the cortical hand movement-related areas, and provide information that can facilitate closing the gap between ESI and BCIs.

## Simulation of source activity in the hand movement-related areas

To simulate activity we made use of the EEG forward equation that defines the EEG:

1 $$\begin{aligned} {{\varvec{y}}} = {{\varvec{M}}}{{\varvec{x}}} + \varepsilon \end{aligned}$$

where the matrix ${{\varvec{y}}}$ represents the EEG channel data. The matrix ${{\varvec{x}}}$ represents the time courses of the source activity. The matrix ${{\varvec{M}}}$, often called the lead field matrix, represents the morphology and conductivity of the brain and contains the linear relationship between the cortical sources and the signals at the scalp. The matrix $\varepsilon$ represents the noise registered in the measurements. We followed these steps for the simulations: forward modeling, ROI definition, simulation of source time courses, EEG computation, and noise addition.

### Forward modeling

To obtain the lead field matrix ${{\varvec{M}}}$, we computed a boundary element method (BEM) model based on the MRI images of a 27-year-old subject. The MRI images were processed and segmented using Freesurfer [4], and the BEM surfaces of the scalp, skull, and brain were generated using Freesurfer and MNE-python [8]. A set of 339 electrodes named and positioned according to the international 10-05 system were co-registered and projected into the scalp. Then, the lead field matrix for the 10-05 set was computed using the BEM surfaces and the projected electrodes. The number of sources was defined as 4098 per hemisphere, and the default MNE-python conductivities of 0.3, 0.006, and S/m were used for scalp, skull, and brain, respectively.

**Fig. 1:**
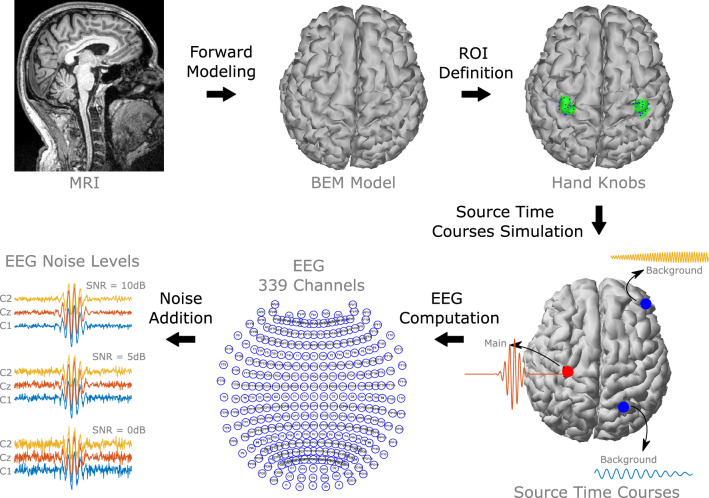
Simulation procedure of the source activity in the hand movement-related areas

### ROI definition

Previous studies [1, 3, 10, 31] have identified the sensory-motor cortex as the source regions where the upper limp movements take place, in particular, the so-called *hand knob* of the precentral gyrus has been found common across these studies. To define the ROI in the hand knobs we inspected the 3D surface of the cortex and manually labeled the center of the hand knob in each hemisphere. Two sets of sources around the markers were established by selecting the 20 closest sources to each marker. The 40 source locations and hand knobs ROIs are depicted in Fig. 1.

### Simulation of source time-courses

Two epochs of 2Hz were simulated per each source in the ROIs, resulting in 80 epochs. In each epoch three sources were activated: the main source within the ROIs and two more background sources outside them. The sources were generated using a sinusoidal Gaussian windowed activity as in [26, 27], by using the following equation:

2 $$\begin{aligned} x_i(t)=a_ie^{-\frac{1}{2}(\frac{t-c_i}{\sigma })^2}sin(2\pi f_it) \end{aligned}$$

The time course of the $i-th$ source is defined by the maximum amplitude $a_i$, the time center $c_i$, frequency $f_i$, and window width $\sigma _i$. The three activities were centered at 1Hz. The main source was simulated with a frequency of 10Hz and width 0.12. The background sources were simulated outside the ROIs to emulate brain activity from other areas and generate interference to the ESI algorithms, their location was randomly selected and they should be at least 3cm from the main source. Their amplitude was 10% of the amplitude of the main source, with a width of 0.12 and frequencies of 5 and 20Hz.

### EEG computation and noise addition

The EEG was computed using the forward equation presented in Eq 1, and the matrices ${{\varvec{M}}}$ and ${{\varvec{x}}}$ generated at forward modeling and source time courses simulation stages. After obtaining the matrix ${{\varvec{y}}}$, Gaussian noise was added to represent the noise in the measurements, three different levels of signal-to-noise ratio were used 10, 5, and 0dB.

Figure 1 summarizes the procedure of simulation of source activity in the hand movement-related areas

## Optimal selection of EEG channels

To select and reduce the number of channels, we used the automatic methodology for electrode selection presented in [26]. In it, the non-dominated sorting genetic algorithm II (NSGA-II) is combined with ESI algorithms. The number of channels used during ESI and the localization error are minimized in a multi-objective optimization problem.

### Algorithm modification

In the original work [26], authors applied the methodology over epochs, therefore combinations of channels were optimized in each epoch. In this work, we introduced a main modification: the optimization is performed over all epochs to obtain a single combination instead of an epoch-wise combination.

### Constraints

We performed multiple tests in an attempt to identify combinations that lead to the lower reconstruction errors: constraining the search space to the 10-10 standard electrode placement, without search space constraint, adding a symmetricity constraint to maintain the number of channels equal between both hemispheres and performed cascade search optimization. In the cascade search, we performed three nested optimizations for 32, 16, and 8 channels, the second and third optimization were constrained to the previous combination found.

### Channel optimization process

**Fig. 2:**
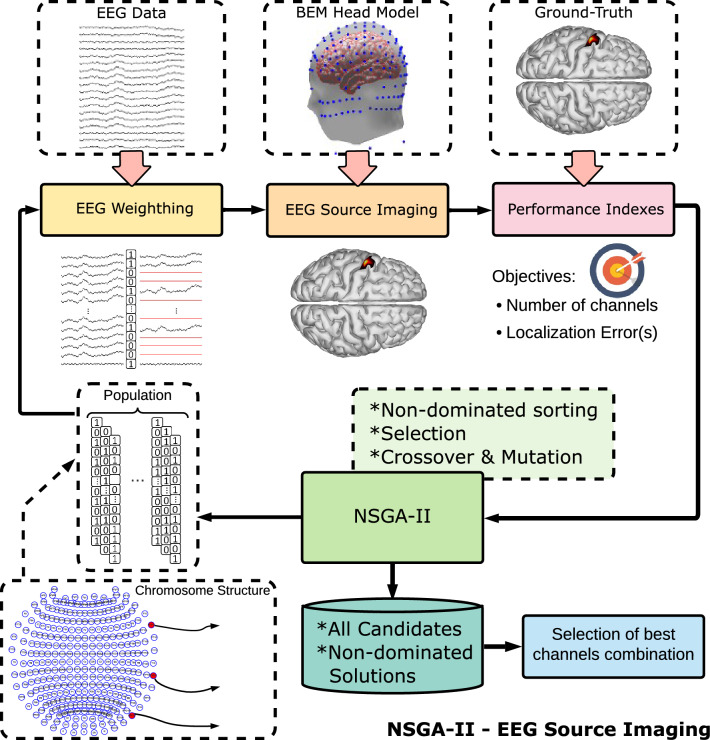
EEG NSGA-II Channel Optimization for ESI

The NSGA-II overall process for ESI is presented in Fig. 2. It starts with an initialization by generating a population of individuals randomly, each individual is represented by a binary chromosome with *g* genes. In the context of channel optimization, each chromosome represents a combination of channels, in which each gene represents a specific channel location as ilustrated in Fig. 2. In a next step, each chromosome is used to weight the EEG. This represents that if a gene has a value of 1, the channel information will be used during ESI, otherwise the channel is not used and its information is zeroing. Then, each individual (combination of channels) of the population is used to perform ESI over each simulated trial. Each individual is evaluated with two performance indexes, objectives to minimize: The average localization error over all trials by comparing the estimated source with the ground truth, and the number of channels. After evaluating each channel combination the performance indexes return to the NSGA-II block. In it individuals are sorted according to their performance using a non-dominated strategy. An individual is said to dominate another if it is superior in at least one objective and not worse in any other objective. In this block, half of the population is selected to continue in the next generation and used to create half of a new generation using crossover and mutation procedures. At this point, each individual is verified to comply with the constraints, otherwise, the individual is removed and a new individual is created, until complete a new generation. This process is repeated with the next generation until a maximum number of generations is reached. After all the process is complete, all the individuals are analyzed to identify the best channel combinations per each number of channels and the non-dominated individuals.

### ESI algorithms

The standardized low-resolution electromagnetic tomography (sLORETA) [20] and weighted minimum norm estimation (wMNE) [6] were used to estimate the source activity during NSGA-II optimization. These algorithms were selected based on the results of previous work in [25, 26], where multiple ESI algorithms were evaluated in ldEEG conditions, and it was found that wMNE and sLORETA consistently obtained the lowest source localization errors. Both algorithms are based on minimum norm estimation, where the ESI problem can be considered as an optimization problem as follows:

3 $$\begin{aligned} {{\varvec{J}}} = argmin_{({{\varvec{x}}})} \{||{{\varvec{M}}}{{\varvec{x}}} - {{\varvec{y}}}||^2_2 \} \end{aligned}$$

As the number of variables to estimate (source activity ${{\varvec{x}}}$) is much higher than the number of observations (EEG channels ${{\varvec{y}}}$) the problem is mathematically ill-posed and ill-conditioned [11]. This means that infinite solutions for the source activity ${{\varvec{x}}}$ can be found to minimize ${{\varvec{J}}}$ and fit with the EEG data ${{\varvec{y}}}$. To find a unique solution, the algorithms make use of Tikhonov-Phillips regularization by including a regularization parameter $\lambda$ that weights the norm of the estimated solution:

4 $$\begin{aligned} {{\varvec{J}}} = argmin_{({{\varvec{x}}},\lambda )} \{ ||{{\varvec{M}}}{{\varvec{x}}} - {{\varvec{y}}}||^2_2 + \lambda ^2||{{\varvec{x}}}||^2_2\} \end{aligned}$$

The ESI solutions of wMNE and sLORETA are given by the following equations:

5 $$\begin{aligned}{} & {} {{\varvec{{\hat{x}}}}}_{wMNE}={{\varvec{W}}}^{-1}{{\varvec{M}}}^{T}({{\varvec{M}}}{{\varvec{W}}}^{-1}{{\varvec{M}}}^{T} + \lambda ^2 I)^{-1}{{\varvec{y}}} \end{aligned}$$

6 $$\begin{aligned}{} & {} {{\varvec{{\hat{x}}}}}_{sLOR}= \sqrt{\frac{1}{[{{\varvec{S}}}_x]_{ii}}} {{\varvec{M}}}^{T}({{\varvec{M}}}{{\varvec{M}}}^{T} + \lambda ^2 I)^{-1}{{\varvec{y}}} \end{aligned}$$

The solution of wMNE uses a weighting matrix ${{\varvec{W}}}$ to influence the weight of the deep sources, resulting in a better localization of the source activity of the deeper sources [5]. Its value is computed using the following equation:

7 $$\begin{aligned} {{\varvec{W}}}^{-1} = diag\left[ \frac{1}{\Vert l_1\Vert _2}, \frac{1}{\Vert l_2\Vert _2}, ...,\frac{1}{\Vert l_s\Vert _2}\right] \end{aligned}$$

where ${{\varvec{W}}}$ is a diagonal matrix, and $\Vert l_s\Vert _2$ the Euclidean norm of the s-th column of ${{\varvec{M}}}$.

The solution of sLORETA is usually smooth (estimations are blurry and widespread over large areas) but it is recognized by its zero localization error in the absence of noise [20]. In its solution sLORETA introduces a non-linear standardization of the solution using the variance of the estimated activity ${{\varvec{S}}}_x$, this variance is defined by:

8 $$\begin{aligned} {{\varvec{S}}}_x={{\varvec{M}}}^{T}({{\varvec{M}}}{{\varvec{M}}}^{T} + \lambda ^2 I)^{-1}{{\varvec{M}}} \end{aligned}$$

The Eucledian distance was used to compute the localization error by comparing the position of the ground-truth source $P_x$ and the estimated source position $P_{{\hat{x}}}$ using the follow equation:

9 $$\begin{aligned} LocE = \Vert P_x-P_{{\hat{x}}}\Vert _2 \end{aligned}$$

where $P_{{\hat{x}}}$ is selected from the estimated source activity ${{\varvec{{\hat{x}}}}}$ by selecting the location of the source with the highest power value.

## Classification of motor imagery task using selected optimal channels and ESI

In order to evaluate the optimal channels selected in the previous step, the same participant who underwent the MRI session, and whose brain model was used for the channel optimization, was invited to participate in an EEG recording session while performing a protocol of motor imagery for hands movements.

### Data recording

**Fig. 3:**
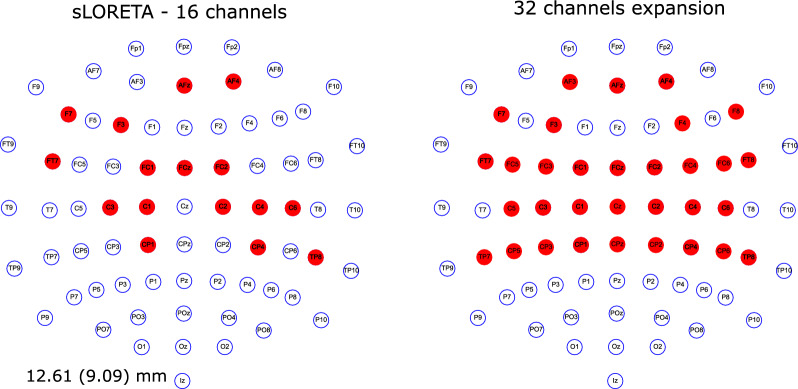
Combinations of 16 optimal channels for sLORETA constrained to 10-10 system and with cascade search, and the 32 channels used for recording

The motor activity was recorded with a 32-channel EEG amplifier (Explore$+$, Mentalab GmbH, Munich, Germany). A cap based on the standard 10-10 was used to attach the electrodes to the scalp and a wet electrode was attached to the earlobe as a reference for the amplifier. The position of the electrodes was defined based on the results of Section 3, by constraining the search space to electrode positions within the 10-10 system and performing a cascade search. To compare optimal channels versus a symmetric distribution covering mostly the sensory-motor cortex, the optimal 16 electrodes with sLORETA (localization error of 12.61mm, see last column in Table 1) were selected. To obtain the 32 channels, the optimal distribution with 16 was expanded by adding the corresponding electrodes in the opposite hemisphere and below and above the Cz until obtaining the most symmetrical distribution possible, Fig. 3 presents the 16 optimal selected electrodes (constrained 10-10 system and cascade search) and their expansion to make a symmetrical distribution of 32 channels. Dry electrodes were used during the data collection, the impedances were measured before the beginning of the recordings and they were kept below 50k$\Omega$.

### Motor imagery/execution protocol

**Fig. 4:**
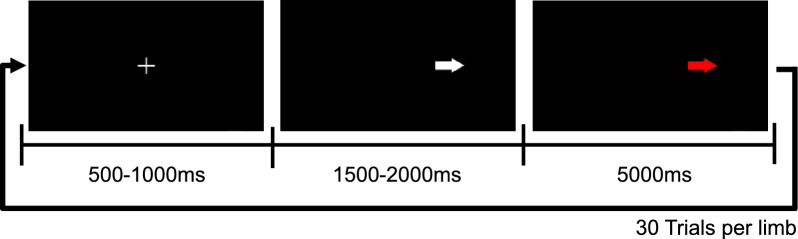
Protocol of goal-directed motor imagery/executed movement, an example of a right hand trial is displayed

A modified version of a goal-directed protocol from [21] was implemented. In each trial, a fixation cross is presented during 500–1000 mHz. Then, a white arrow was shown during 1500–2000 mHz to the participant either at the right or left side of the screen to indicate the goal to touch and the hand to use. Finally, the arrow changed color to red as a cue to start imagining/executing the movement to touch the arrow on the screen, the cue lasts for 5000 mHz. Two runs for each motor execution and motor imagery were recorded. In each run, 30 trials were performed for each hand. A run for execution was performed before each imagination run to facilitate the imagination of the task. Within each run the trials were randomized to avoid carryover effects from one trial to the next. Figure 4 presents an example of the implemented protocol.

### Data processing

The data processing and classification have been done offline, after the recording the data was imported and processed using MNE-python [8]. A zero-phase finite impulse response (FIR) notch filter was applied to remove the power line at 50 and 100 Hz. Then a zero-phase FIR high-pass filter is applied at 0.1 Hz. The channels are divided into two sets at this point by forming a set containing all the 32 channels, and a set containing the 16 optimal channels. A common average reference (CAR) procedure is applied to each set of channels, the channels were split into two groups to emulate the recording with only 16 channels and avoid any influence of the complementary 16 channels over the optimal 16 channels during the CAR procedure. Then, a band-pass filtered with cutoff frequencies of 8 and 12 Hz was applied to each set of data to extract the activity in the frequency of the Mu rhythm [22, 31]. Finally, epochs of each trial were defined between − 1000 and 3000 mHz before and after the cue to start imagining/executing the limb.

### Feature extraction and classification

Two types of epochs are used as input for the feature extraction process: The epochs directly from electrode data and the epochs from the estimated sources. The epochs in the source space are computed using sLORETA. The ESI method computes the activity of the whole set of 8196 sources and then the activity of the handknobs ROI is extracted using the same ROI criterion as in Sect. 2, extracting only the time courses of the 40 selected locations in the handknobs ROI. The epoched data from source/electrode space is used as input for feature extraction performed by applying the common spatial filter (CSP) method. To apply the CSP, the data is split into testing (30%) and training (70%), and the CSP is computed in the training set and then applied to the testing set. Similarly, the features are standardized by computing and applying a standard scaler over the training features and then applying it over the testing features. Then the training features are used to train three models based on the following types of classifiers: Random Forest (RF), support vector machine (SVM), and linear discriminant analysis (LDA). Finally, the trained models are evaluated over the unseen testing features. This process is repeated 10 times to form a 10-fold cross-validation. During each fold, the accuracy, F1 score, precision, and recall are computed from the confusion matrix, where the true negative (TN) and true positive (TP) predictions are presented in the diagonal, and the false negative (FN) and false positive (FP) predictions in the anti-diagonal, the metrics are computed using the following equations:

10 $$\begin{aligned}{} & {} \text {Accuracy} = \frac{TN + TP}{TN + FN + TP + FP} \end{aligned}$$

11 $$\begin{aligned}{} & {} \text {Precision} = \frac{TP}{TP + FP} \end{aligned}$$

12 $$\begin{aligned}{} & {} \text {Recall} = \frac{TP}{TP + FN} \end{aligned}$$

13 $$\begin{aligned}{} & {} \text {F1 score} = 2 \cdot \frac{precision \cdot recall}{precision+recall} = 2 \cdot \frac{TP}{2TP + FP + FN} \end{aligned}$$

## Results

### Optimal channel selection

A summary of the performed tests for the optimal selection of EEG channels is presented in Table 1. The localization error presented is the mean of the localization error across all epochs. We first evaluated the dataset with the three levels of added noise and constrained the search space to the 10–10 standard electrode placement. The localization error between the three levels of noise was similar, i.e. for 8 channels the errors were between 15.45 and 16.07 mm for sLORETA and 16.08–17.01 mm for wMNE. As the difference is less than 1mm between the highest and lowest error for all electrode counts, we decided to continue the evaluations only with the dataset of higher noise level (0dB).

**Table 1:** Localization error (mm) and standard deviation of the optimization test

	Dataset	10dB	5dB	0dB				
	Constraint type	10–10 system			10–10 system, symmetricity, cascade search	Symmetricity	No constraints	10–10 system, cascade search
8 chs	sLORETA	15.87 (10.54)	15.45 (9.03)	16.07 (10.11)	18.59 (7.97)	**14.29 (5.04)**	14.74 (7.24)	16.75 (11.68)
	wMNE	16.08 (8.87)	16.19 (9.22)	17.01 (13.05)	19.49 (10.69)	14.94 (7.69)	**14.80 (10.84)**	16.64 (12.12)
16 chs	sLORETA	12.66 (9.10)	12.58 (9.04)	13.11 (8.77)	16.45 (9.58)	11.56 (5.57)	**10.69 (6.61)**	12.61 (9.09)
	wMNE	12.90 (8.92)	13.62 (8.82)	13.64 (8.89)	15.65 (8.87)	12.63 (5.11)	**12.01 (5.74)**	13.91 (9.19)
32 chs	sLORETA	7.80 (8.70)	7.85 (8.42)	7.74 (8.68)	8.42 (8.81)	6.02 (7.38)	**5.07 (5.64)**	8.41 (8.49)
	wMNE	7.30 (8.74)	7.32 (8.31)	6.45 (7.82)	7.20 (8.39)	**3.99 (6.37)**	5.18 (6.29)	6.62 (8.60)
72 chs (10-10 system)	sLORETA	**4.00 (7.15)**	4.01 (7.15)	4.05 (7.16)	4.05 (7.16)	–	–	4.05 (7.16)
	wMNE	3.77 (6.99)	3.75 (6.98)	**3.65 (6.99)**	**3.65 (6.99)**	–	–	**3.65 (6.99)**
339 chs	sLORETA	–	–	–	–	**0.00 (0.00)**	**0.00 (0.00)**	–
	wMNE	–	–	–	–	**0.00 (0.00)**	**0.00 (0.00)**	–

The values remarked correspond to the best result with a given number of channels and ESI method. The values in the parenthesis correspond to the standard deviation

**Fig. 5:**
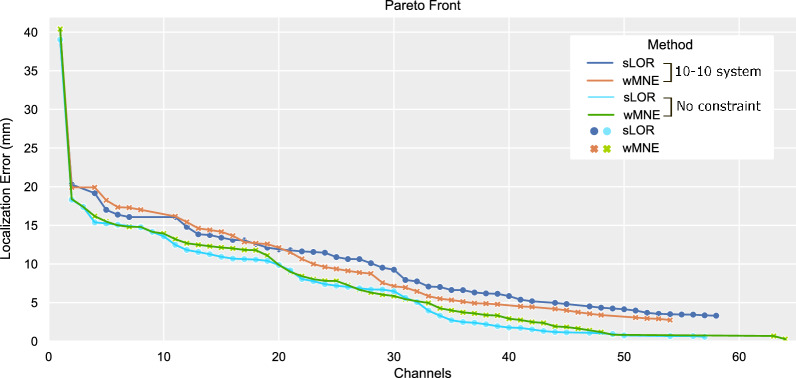
Comparison of Pareto fronts for SNR 0dB dataset when constraining the search space to the 10–10 positioning system and without constraining (search space of 339 positions)

The less accurate results were obtained when adding multiple constraints, in particular, the case when the optimization was performed in cascade with hemispherical symmetricity and search within the 10–10 system. The effect of these constraints increased the localization error between 2.01 and 3.34mm in the lower channel counts of 8 and 16 channels when compared with only applying the 10–10 system constraint. On the contrary, when fewer constraints were imposed, the accuracy increased. As shown in Table 1, the highest accuracy values were obtained when no constraint was imposed or when only applying symmetricity constraint. These results coincide with the bigger search space of 339 channels, as no 10-10 system constraint was imposed in both cases. In these two cases, the localization error was lowered between 1.63 and 2.67 mm when compared with the 10–10 system constraint. The Pareto fronts when constraining the search space to 10–10 system and without constraint, search space of 339 channels, are presented in Fig. 5. It is noticeable that the Pareto fronts of sLORETA and wMNE were more accurate when not limiting the search space.

**Fig. 6:**
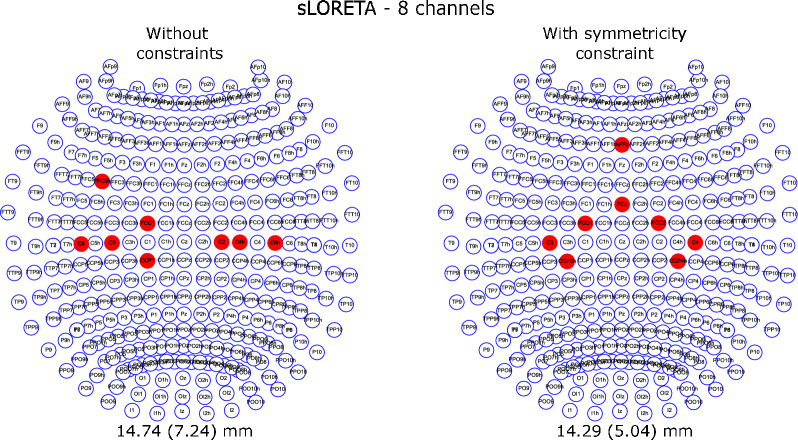
Combinations of 8 channels for sLORETA without constraints and with symmetricity constraint

The 8 channel combinations for the cases with the search space of 339 channels with and without symmetricity for sLORETA are presented in Fig. 6. From them, it can be seen that the electrodes were found close to the motor cortex areas, in both cases with one electrode slightly separate from the others.

### Classification

A summary of the classification results is presented in Table 2. The results are divided into electrode space and source space. When comparing the results in those domains it can be seen that the classification metrics are higher when extracting features from the handknobs source space than the classification metrics from the same classifier and number of channels when extracting features from the electrode space. Notice that only for one case the performance was better in the electrode space (16 channels, RF classifier). In addition when comparing the accuracy between the number of channels, in all cases except one, the performance was higher with the optimally selected 16 channels than the 32 typical electrode distribution. The highest classification accuracy 0.8444(0.0499) was obtained using a LDA classifier extracting the features from reconstructed source space. The reconstruction was obtained by combining ESI with sLORETA and the 16 optimally selected electrodes.

**Table 2:** Classification accuracy, F1-score, Precision, and Recall

Electrode space					
No Chs.	Classifier	Accuracy	F1 Score	Precision	Recall
32	SVM	0.7195 (0.0672)	0,712 (0,0710)	0,7372 (0,0658)	0,7265 (0,0609)
32	RF	0.725 (0.0473)	0,7223 (0,0484)	0,731 (0,0458)	0,7327 (0,0456)
32	LDA	0.6972 (0.0576)	0,6949 (0,0587)	0,706 (0,0582)	0,7075 (0,0590)
16	SVM	0.7667 (0.0768)	0,7629 (0,0786)	0,7764 (0,0687)	0,7731 (0,0698)
16	RF	**0.8167 (0.0586)**	**0,8155 (0,0588)**	**0,8208 (0,0558)**	**0,8244 (0,0578)**
16	LDA	0.7694 (0.0569)	0,764 (0,0591)	0,7778 (0,0624)	0,7673 (0,0572)

Source space					
No Chs.	Classifier	Accuracy	F1 Score	Precision	Recall
32	SVM	0.7387 (0.0598)	0,7374 (0,0595)	0,7504 (0,0613)	0,7471 (0,0610)
32	RF	0.7667 (0.0544)	0,7644 (0,0548)	0,7719 (0,0571)	0,7695 (0,0541)
32	LDA	0.7054 (0.0377)	0,7029 (0,0370)	0,7067 (0,0340)	0,7103 (0,0396)
16	SVM	0.8083 (0.0576)	0,8068 (0,0579)	0,8173 (0,0544)	0,815 (0,0528)
16	RF	0.7194 (0.0761)	0,7168 (0,0771)	0,7218 (0,0796)	0,7238 (0,0793)
16	LDA	**0.8444 (0.0499)**	**0,8421 (0,0520)**	**0,8459 (0,0559)**	**0,8485 (0,0481)**

The metrics were obtained by extracting features from electrode data and extracting features from reconstructed sources by sLORETA. The values remarked correspond to the best result in Source and Electrode Space. The values in the parenthesis correspond to the standard deviation

## Discussion and conclusion

The localization error is an indication of the spatial accuracy, here, in the best evaluation cases we obtained 14.29 mm (8 channels, sLORETA, and only symmetricity constraint), 10.69 mm (16 channels, sLORETA, and no constraints), and 3.99 mm (32 channels, wMNE, and only symmetricity constraint). As in [27], we confirmed that the channel optimization with NSGA-II enables us to find channel combinations that led to the closest values to hdEEG accuracy values, in particular, the combination with 32 channels is less than 0.5 mm from the accuracy obtained with 72 channels in 10–10 system.

This research provides a pipeline to optimize the number of channels and identify ldEEG channel combinations for an individual subject that reduces the gap between hdEEG and ldEEG spatial accuracy. This systematic search for the best electrode positions was done as a first step in the design of dedicated EEG systems that can monitor the cortical source activity and facilitate the implementation of BCI systems for assisting in the rehabilitation of hand movement in stroke survivors. The previous studies in [3, 7, 14, 28] demonstrated that the source space can outperform the sensor space. Here, our results indicate that 16 channels could provide an accurate reconstruction to be used in BCIs related to hand movements.

Previous works have applied NSGA optimization to improve classification task in emotion studies [18], EEG sleep scoring [29], and EEG epilepsy detection [19]. However those studies focused on the electrode space, while in here, the channel optimization pipeline was applied to estimate the activity in ROIs with ESI, and then the optimized channels were used in a BCI context for motor imagery classification. It is important to indicate that the optimized channels have been found using the head model of the specific participant. We anticipate that optimal channels can vary across subjects due to inter-subject variability, and across neuroparadigms as the brain ROI change according to the activity to study. However, further studies should be done to evaluate the optimized channels with template models over large subjects populations, and clarify the impact of combining ESI with channel optimization in other classification tasks.

The level of accuracy required for source-based BCIs for hand movements might depend on the type of imagined movements to classify. The boundaries of the applications should be clarified in further studies, i.e. it is noticeable that classifying between right or left hand might require lower spatial accuracy than classifying within wrist movements of the same limb. As shown here, the spatial accuracy of the 16 optimal channels was enough the obtain better performance than with 32 channels, and also enough to obtain better performance when using the source space for classification rather than the electrode space.

The results indicate that when a bigger search space is used, better localization accuracy could be obtained. This should be considered in BCI systems, exploring electrode locations outside the standard positioning systems towards a personalized set of combinations can be valid in a BCI context if it leads to a better classification, future works should explore individual channel distributions and their classification performance. Here, we demonstrated that the use of electrode locations outside the standard led to lower reconstruction errors.

This study focused specifically on hand movements, restricting the ROIs to the hand knobs. It is worth noting that similar procedures could be applied to other limbs or different regions of the brain, such as estimating the source activity in areas related to hearing, vision, or attention. To the best of our knowledge, no other studies have been conducted to evaluate the ESI properties on particular brain regions using ldEEG with optimized channel selection, and this framework can be generalized to particular ROIs. Here, the EEG simulation was limited to sinusoidal Gaussian activity and this may not fully capture the complex behavior of a real EEG recording. However, the simulation framework serves as a basis to evaluate the spatial accuracy in the context of ldEEG source imaging, considering that the reduced spatial sampling has been one of the arguments against the use of source-estimated activity in BCI systems. It is debatable whether increasing the complexity of the simulated signal will affect the spatial resolution, especially when considering that the non-linear mix imposed by the volume conduction has been included during forward modeling. Another limitation is that the motor classification task results are based on a single participant’s data. However, it is noticeable the impact of modeling and optimizing the number of electrodes, and the fact that the data was recorded based on the results of the optimization, demonstrated that for the single participant, the classification was better when using the optimal set than when using all the channels, and moreover, that the highest classification was obtained extracting features for the reconstructed sources of the same handknobs ROI that was used during simulation.

The ESI estimations were performed with well-known methods wMNE and sLORETA. However, recent studies suggest, that multimodal approaches [13] and deep learning algorithms [12, 30] can be beneficial for the estimation of source activity by improving the spatiotemporal accuracy. Further studies, including these novel approaches, should be done to comprehensively evaluate their potential for BCI applications.

In conclusion, this study explores the use of optimized ldEEG for estimating the source activity of the hand movement related areas and investigates the accuracy under multiple optimization scenarios. In this work, several key findings are reported. Firstly, optimized channel selection in ldEEG setups demonstrated potential as a viable alternative to hdEEG, offering a comparable accuracy when retrieving the source space of the particular ROI. This finding is significant as it paves the way for source-centered BCI systems with low EEG channel counts.

Moreover, we presented a comprehensive pipeline to perform channel optimization in the context of ESI. The pipeline can be used to identify the channels that can accurately estimate the sources in a ROI and to be used in developing customized EEG solutions for a particular user when using individual MRI for forward modeling. As demonstrated for the participant, the results of the pipeline indicate than a higher classification accuracy can be obtained from the selected channels in electrode and source space.

Furthermore, as a result of the reduction of channels, the optimized ldEEG can improve the practicality of EEG in real-world scenarios, as fewer sensors often lead to wearable and more easy-to-use devices. For instance, if a person wears a EEG headset to operate a BCI daily, if a equal or better performance can be achieved with lower electrodes, then it would be easier to set-up less channels and less costly to acquire the technology. This principle can be applied to various scenarios, including clinical applications such as neurorehabilitation following stroke, monitoring attention in ADHD patients, and tracking specific brain regions associated with conditions like depression, anxiety, epilepsy, and sleep disorders. Further studies are needed to explore the potential impact of source-centered channel optimization in these applications. It can be argued that the estimation of the sources increases computational complexity, especially for online systems. However, pre-calculated forward models and inverse operators can serve to speed up the computations.

This work provides insights into the use of optimized ldEEG in retrieving sources towards BCI systems. Including an evaluation on a single subject of how ESI improved the classification performance in a motor imagery task. However, several questions are still open and are required to be solved prior to a larger implementation in BCI systems. Further studies should be performed to clarify the role of ESI with optimized sensors and to develop source-centered BCIs that can complement current BCI systems based on only scalp recordings. Also, to analyze the effect of optimized channels in the classification accuracy when using source and sensor space in larger datasets. Further efforts should be made to verify the implications of the source computation in online settings and clarify whether applying forward modeling on an individual basis or using brain structural information from template heads can be accurate enough.

## Data Availability

We acknowledge the importance of data transparency and are committed to providing access upon request to the anonymized MRI, simulated EEG data, and recorded EEG data.

## References

[1] Bradberry TJ, Gentili RJ, Contreras-Vidal JL (2010). Reconstructing three-dimensional hand movements from noninvasive electroencephalographic signals. Journal of Neuroscience.

[2] Cincotti F, Mattia D, Aloise F, Bufalari S, Astolfi L, Fallani FDV, Tocci A, Bianchi L, Marciani MG, Gao S, Millan J, Babiloni F (2008). High-resolution eeg techniques for brain-computer interface applications. Journal of Neuroscience Methods.

[3] Edelman BJ, Baxter B, He B (2016). Eeg source imaging enhances the decoding of complex right-hand motor imagery tasks. IEEE Transactions on Biomedical Engineering.

[4] Fischl B (2012). FreeSurfer. NeuroImage.

[5] Fuchs M, Wagner M, Köhler T, Wischmann HA (1999). Linear and nonlinear current density reconstructions..

[6] Fuchs M, Wagner M, Wischmann HA (1994). Generalized minimum norm least squares reconstruction algorithmss. ISBET Newsletter.

[7] Giri A, Kumar L, Gandhi T (2021). Cortical source domain based motor imagery and motor execution framework for enhanced brain computer interface applications. IEEE Sensors Letters.

[8] Gramfort A, Luessi M, Larson E, Engemann DA, Strohmeier D, Brodbeck C, Goj R, Jas M, Brooks T, Parkkonen L, Hämäläinen M (2013). Meg and eeg data analysis with mne-python. Frontiers in Neuroscience.

[9] Hardwick RM, Caspers S, Eickhoff S, Swinnen SP (2018). Neural correlates of action: comparing meta-analyses of imagery, observation, and execution. Neurosci Biobehav Rev.

[10] He B, Baxter B, Edelman BJ, Cline CC, Ye WW (2015). Noninvasive brain-computer interfaces based on sensorimotor rhythms. Proc IEEE.

[11] He B, Sohrabpour A, Brown E, Liu Z (2018). Electrophysiological source imaging: a noninvasive window to brain dynamics. Ann Rev Biomed Eng.

[12] Jiao M, Wan G, Guo Y, Wang D, Liu H, Xiang J, Liu F (2022). A graph fourier transform based bidirectional long short-term memory neural network for electrophysiological source imaging. Front Neurosci.

[13] Jiao M, Yang S, Wang B, Xian X, Semenov YR, Wan G, Liu F (2023). MMDF-ESI: multi-modal deep fusion of EEG and meg for brain source imaging. Lecture Notes Comp Sci.

[14] Li C, Guan H, Huang Z, Chen W, Li J, Zhang S (2021) Improving movement-related cortical potential detection at the eeg source domain. International IEEE/EMBS Conference on Neural Engineering, NER. (5):214–217. 10.1109/NER49283.2021.9441169

[15] Lotte F, Bougrain L, Cichocki A, Clerc M, Congedo M, Rakotomamonjy A, Yger F (2018). A review of classification algorithms for EEG-based brain-computer interfaces: a 10 year update. J Neural Eng.

[16] Mattiocco M, Babiloni F, Mattia D, Bufalari S, Sergio S, Salinari S, Marciani M.G, Cincotti F (2006) Neuroelectrical source imaging of mu rhythm control for bci applications. Annual International Conference of the IEEE Engineering in Medicine and Biology—Proceedings. 980–983. 10.1109/IEMBS.2006.260128 10.1109/IEMBS.2006.260128 17945612

[17] Michel CM, Brunet D (2019). Eeg source imaging: A practical review of the analysis steps. Frontiers in Neurology.

[18] Moctezuma LA, Abe T, Molinas M (2022). Two-dimensional CNN-based distinction of human emotions from EEG channels selected by multi-objective evolutionary algorithm. Sci Reports.

[19] Moctezuma LA, Molinas M (2020). EEG channel-selection method for epileptic-seizure classification based on multi-objective optimization. Front Neurosci.

[20] Pascual-Marqui RD (2002). Standardized low-resolution brain electromagnetic tomography (sLORETA): technical details. Methods Finding Exp Clin Pharmacol.

[21] Pereira J, Ofner P, Schwarz A, Sburlea AI, Müller-Putz GR (2017). EEG neural correlates of goal-directed movement intention. NeuroImage.

[22] Pfurtscheller G, Brunner C, Schlögl A, da Silva FHL (2006). Mu rhythm (de)synchronization and eeg single-trial classification of different motor imagery tasks. NeuroImage.

[23] Pfurtscheller G, Silva FHLD (1999). Event-related EEG/meg synchronization and desynchronization: basic principles. Clin Neurophysiol.

[24] Saha S, Baumert M (2020). Intra- and inter-subject variability in EEG-based sensorimotor brain computer interface: a review. Front Comput Neurosci.

[25] Soler A, Giraldo E, Lundheim L, Molinas M (2022) Relevance-based Channel Selection for EEG Source Reconstruction: An Approach to Identify Low-density Channel Subsets. Proceedings of the 15th International Joint Conference on Biomedical Engineering Systems and Technologies, BIOSTEC 2, BIOIMAGING: 174–183. 10.5220/0010907100003123

[26] Soler A, Moctezuma LA, Giraldo E, Molinas M (2022). Automated methodology for optimal selection of minimum electrode subsets for accurate EEG source estimation based on genetic algorithm optimization. Sci Reports.

[27] Soler A, Muñoz-Gutiérrez PA, Bueno-López M, Giraldo E, Molinas M (2020). Low-density EEG for neural activity reconstruction using multivariate empirical mode decomposition. Front Neurosci.

[28] Srisrisawang N, Müller-Putz GR (2022). Applying dimensionality reduction techniques in source-space electroencephalography via template and magnetic resonance imaging-derived head models to continuously decode hand trajectories. Front Human Neurosci.

[29] Stenwig H, Soler A, Furuki J, Suzuki Y, Abe T, Molinas M (2022) Automatic sleep stage classification with optimized selection of eeg channels. Proceedings - 21st IEEE International Conference on Machine Learning and Applications, ICMLA. 1708–1715 . 10.1109/ICMLA55696.2022.00262

[30] Sun R, Sohrabpour A, Worrell GA, He B (2022). Deep neural networks constrained by neural mass models improve electrophysiological source imaging of spatiotemporal brain dynamics. Proc Natl Acad Sci United States Am.

[31] Yuan H, Doud A, Gururajan A, He B (2008). Cortical imaging of event-related (de)synchronization during online control of brain-computer interface using minimum-norm estimates in frequency domain. IEEE Trans Neural Syst Rehabilit Eng.

